# Supplementary material for: Integration of Visual Information in Auditory Cortex Promotes Auditory Scene Analysis through Multisensory Binding
Source: Neuron. 2018 Feb 7;97(3):640–655.e4. doi: 10.1016/j.neuron.2017.12.034 (PMC5814679; doi:10.1016/j.neuron.2017.12.034)
Supplement: Document S1. Figures S1–S7 [file mmc1.pdf]

Neuron, Volume *97*

Supplemental Information

Integration of Visual Information in Auditory Cortex Promotes Auditory Scene Analysis through Multisensory Binding

Huriye Atilgan, Stephen M. Town, Katherine C. Wood, Gareth P. Jones, Ross K. Maddox, Adrian K.C. Lee, and Jennifer K. Bizley

Supplemental Results

Supplemental Figure 1 (related to Figure 1)

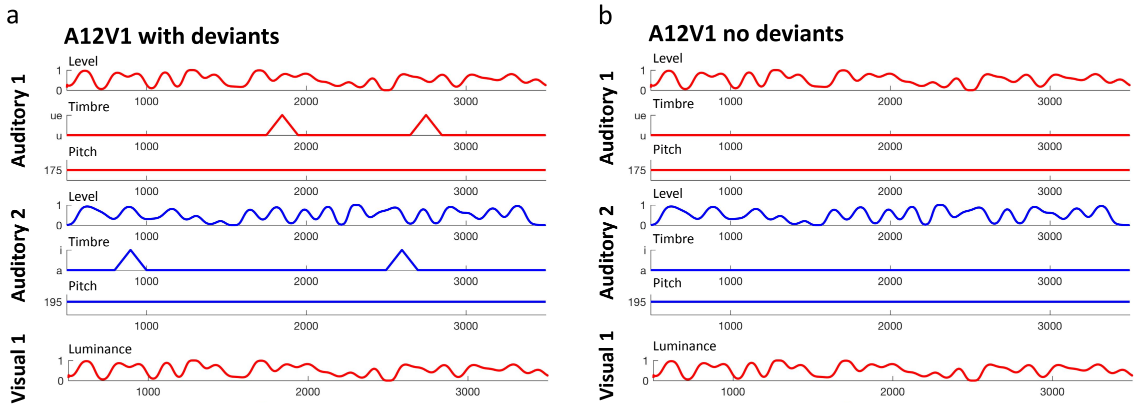

Schematic of the stimuli used in this study to illustrate the difference between stimuli with deviants (a) and without (b). Each row depicts the time course of each feature within the stimulus over a single trial. Importantly, the timing of the timbre deviants is not predicted by the temporally coherent changes in the binding features: here sound level and visual luminance.

Supplemental Figure 2 (related to Figure 2)

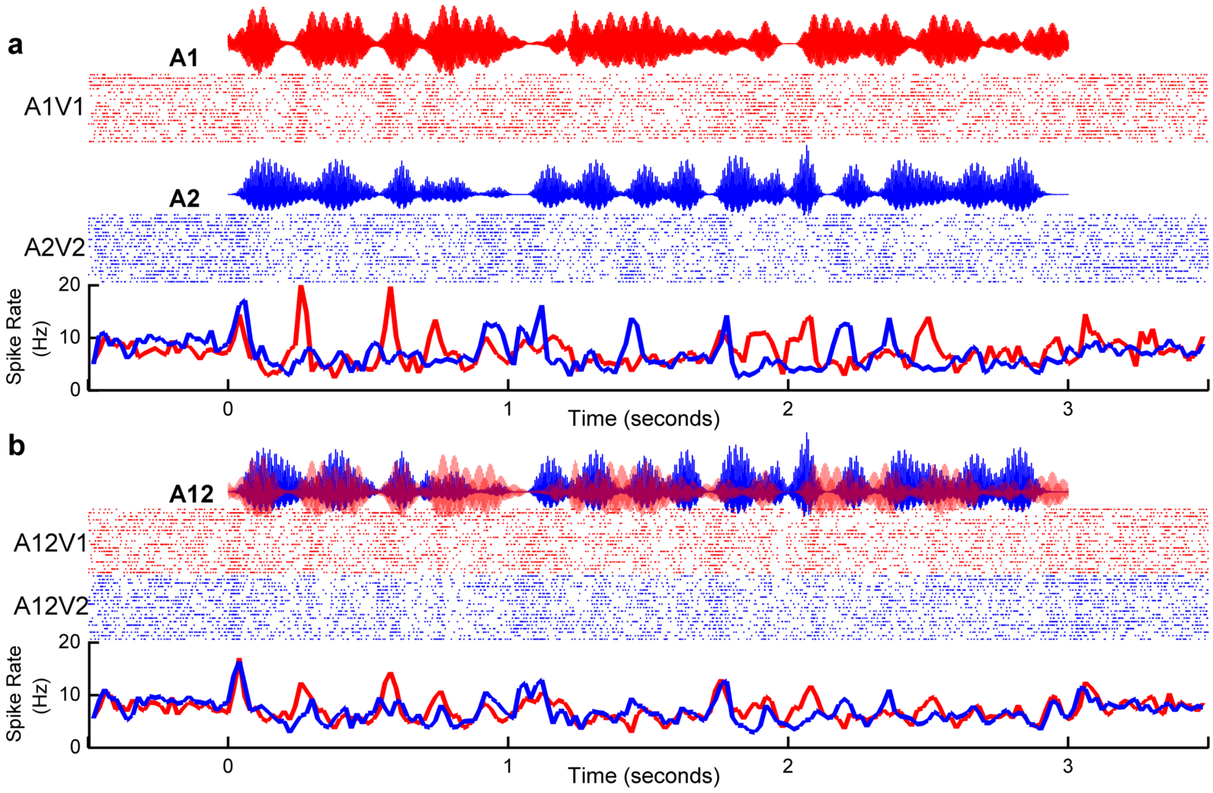

Visual stimuli can determine which sound stream auditory cortical neurons follow in a mixture: example auditory-discriminating unit

The spiking responses of an example unit are shown to coherent single stream (**a**) and dual stream stimuli (**b**). This example unit was an auditory discriminating unit recorded in an awake animal. In this example 68% (15/22) of responses were classified as A1 when the visual stimulus was V1, and 40 % of responses (9/22) were classified as A1 when the visual stimulus was V2, yielding a VPI score of 28%.

Supplemental Figure 3 (related to Figures 2 and 3)

Effects of cortical field, cortical lamina and response type on visual modulation of dual stream responses

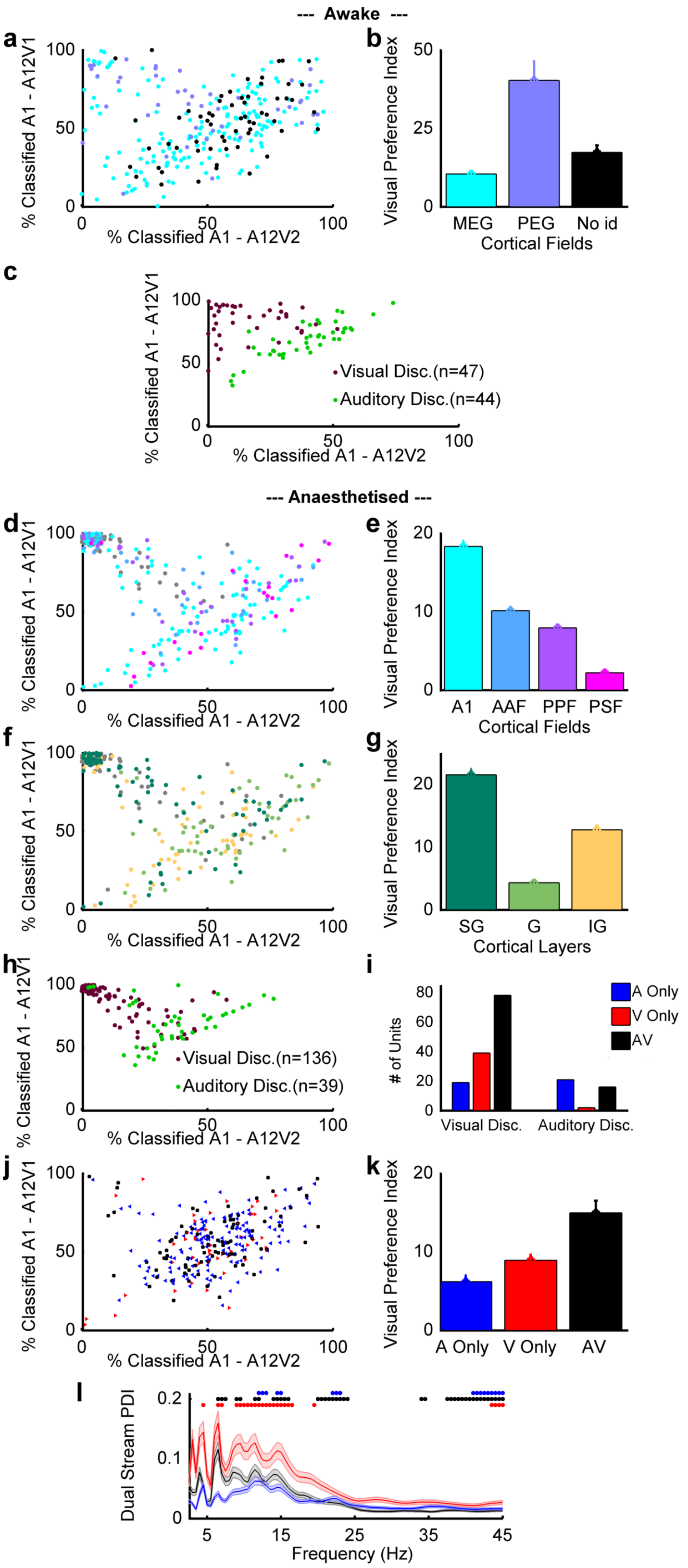

a The distribution of decoding values for dual stream responses, according to the visual condition (as in Figure 2C) colour coded according to recording location. Awake recordings were made from animals in which recording electrodes targeted the MEG (where A1 and AAF are located) and PEG (where fields PSF and PPF are located). The sampling density of our recording arrays (16 electrodes in a 4 x 4 grid with electrodes separated by 800 μm) does not provide the high spatial resolution necessary to determine recording locations precisely – particularly at the low frequency reversal that separates PEG from MEG in the ferret. Therefore some recording electrodes are classified as 'unidentified' as in these cases neither the frequency tuning, nor the post-mortem histology, allowed us to unambiguously ascribe the recording site to MEG or PEG. **b** VPI scores from **a** by cortical area (mean ±SEM). A one way ANOVA revealed there to be a significant effect of field ($F(2,278) = 7.1354$, $p = 9.5072e-04$), with post-hoc comparisons indicating PEG was significantly higher than MEG or unidentified sites. **c** re-plots the data in **a** showing only units with a significant VPI, colour-coded according to whether they were visual classified or auditory classified. **d,e,** as **a,b** but for the anesthetised dataset where we were able to make sufficient penetrations (30-50) to generate a high resolution tonotopic map and hence ascribe recording sites to cortical subfields. A one way ANOVA revealed there to be no significant effect of field on VPI ($F(3,1130) = 2.1886$, $p = 0.0877$).

Recordings in anesthetised animals were made with linear shank electrodes, facilitating current source density analysis to identify the cortical layers. **f** shows the distribution of decoding values in the dual stream condition according to recording location and depth in the anesthetised dataset. **g** summarises the data in **c** by cortical field. A one-way ANOVA across cortical layers showed a significant effect of layer ($F(2,1134) = 3.1543$, $p = 0.0430$) with post-hoc comparisons indicating that the VPI scores were greater in the supra-granular than granular layers. **h** plots the distribution of dual stream decoding values for only units with a significant VPI, colour-coded according to whether they were classified as auditory-discriminating or visual-discriminating. In the anesthetised animal we additionally used simple noise bursts and light flashes to describe units as auditory (A; n=160 units), visual (V, n=53) or auditory visual (AV; grey, n=94). **i** shows the distribution of A, V and AV units that were also classified as auditory-discriminating or visual-discriminating. Of 136 visual discriminating units with a significant VPI, 19 were categorised as auditory, 39 as visual and 78 as auditory-visual, of 39 auditory-discriminating units with significant VPI values 21 were auditory, 2 were visual and 16 were auditory visual Fig. S3i. **j,l,** as **d**,**e,** but with units colour coded according to whether they were classified as A, V or AV with simple stimuli. **k** mean (± SEM) dual stream phase dissimilarity index (PDI) values for recording sites categorised according to the spiking responses recorded there. Symbols indicate the frequencies at which the dual stream PDI index was significant (pairwise t-test, $p<0.001$ with correction). While the phase effects are greatest at the sites where visual activity was recorded, significant dual stream PDI values were observed in all three unit types. In all three cases significant phase coherence was seen at 12Hz, 13.5Hz-14.5Hz and 42.5-44.5Hz. Modulation at 10-12 Hz was only observed at sites in which AV and V responses were recorded.

Supplementary figure 4: Related to Figures 2,3 and 5

The effects of temporal coherence on single stream decoding and of visual identity on dual stream decoding are evident in both single and multi-units.

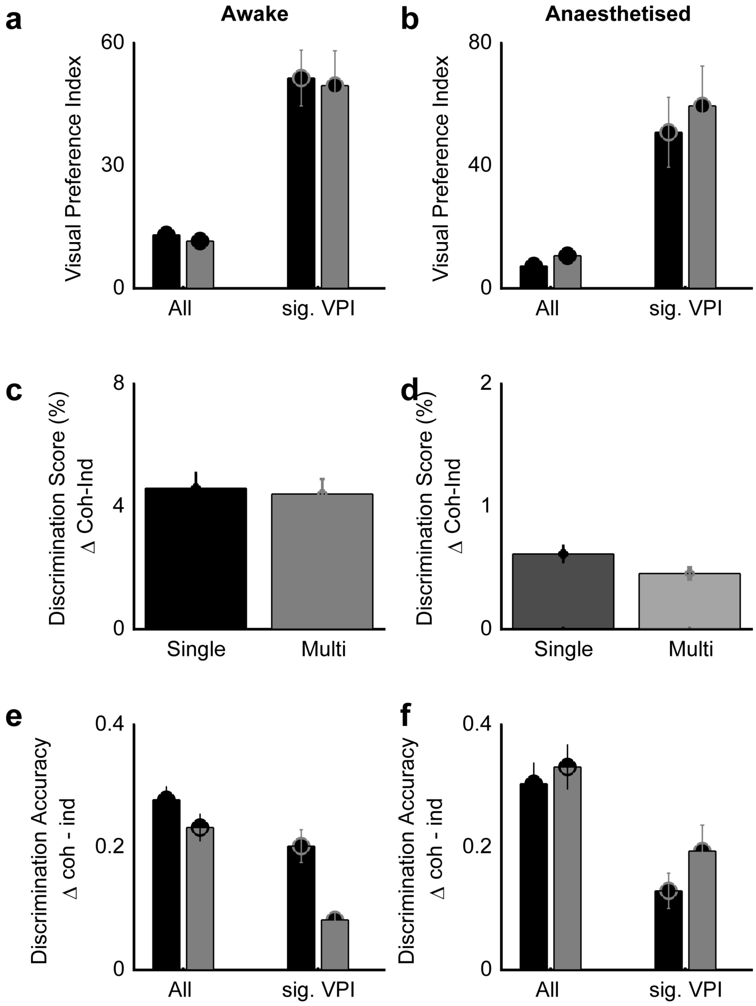

a,b No effect of unit type (single versus multi-unit) was found for VPI values in awake recordings (all units: F(1,270) = 0.160, p = 0.690; significant VPI: F(1,90) = 0.105, p = 0.747) or anaesthetised recordings (all units: F(1,332) = 0.474, p = 0.492; significant VPI: F(1,174) = 0.687, p = 0.412) **c,d** Discrimination scores for timbre deviant detection in dual stream stimuli was indistinguishable for single units and multi units in awake recordings (F(1,167) = 0.033, p = 0.857). and in anaesthetised recordings (F(1,221)=0.834, p = 0.363) .

e,f Single units had significantly higher influence of temporal coherence on discrimination accuracy for single stream stimuli (as in Figure 5e,f) in awake recordings (All units: F(1,270) = 4.992, p = 0.026; for units with significant VPI: F(1,90) = 10.1780, p = 0.0020). Single and multiunits had equivalent performance in the anaesthetised dataset (F (1,332) = 1.256, p = 0.264; significant VPI: F(1,174) = 1.512, p = 0.226).

Supplemental Figure 5, related to Figure 5.

Temporal Coherence did not influence spike rates

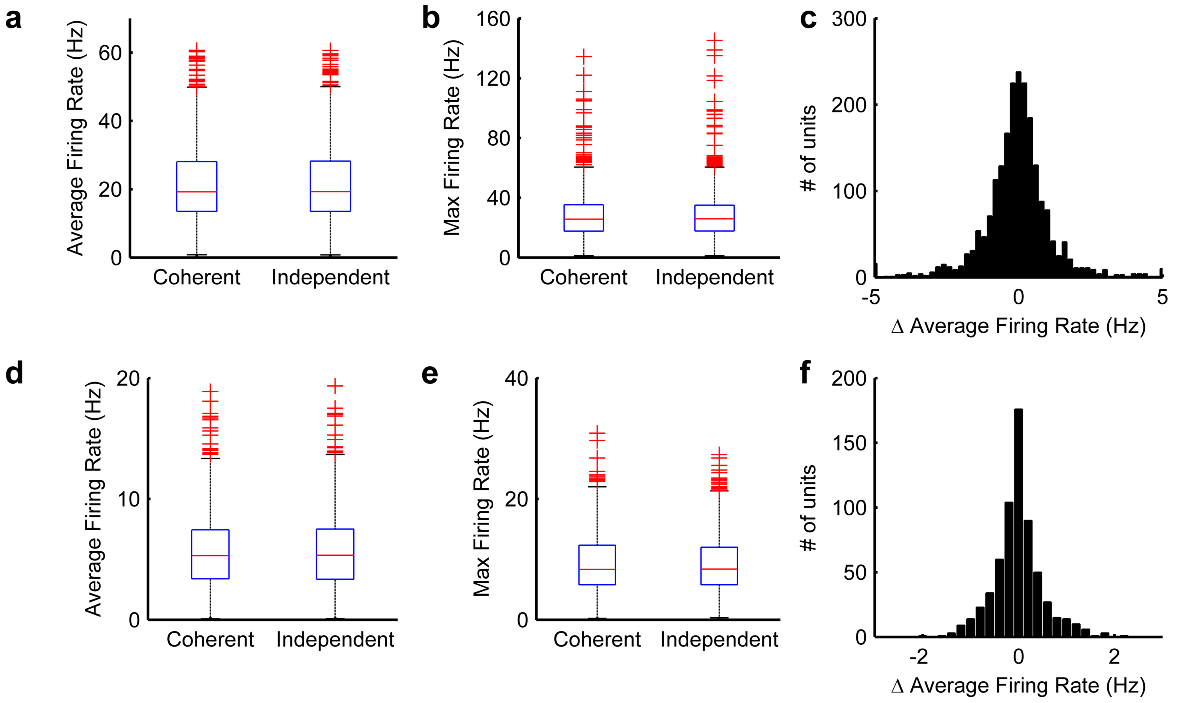

There were no statistically significant changes in mean **(a,d)** or max **(b,e**) firing rate between temporally coherent and temporally independent datasets in either the awake **(a,b,c)** or anesthetised **(d,e,f)** datasets**. Awake dataset:** For all units: Mean firing rate t_{540}= -0.0308, p =0.9754; Max firing rate: t_{540} = 0.4354, p =0.6636. For units with a significant VPI value: Mean: t_{180}= -0.0631, p =0.7694; Max: t_{180}= 0.8563, p =0.0939. **Anesthetised dataset:** all units: Mean firing rate t_{-664} = -0.0638, p = 0.9492. Max firing rate: t_{664}= 0.0047, p =0.9947. Significant VPI units Mean firing rate t_{348} 0.0308, p =0.9498. Max firing rate: t_{348}= 0.0235, p =0.9912.

Supplemental Figure 6 (related to Figure 4).

Temporally coherent visual stimuli enhanced timbre deviant discrimination relative to auditory-only stimuli

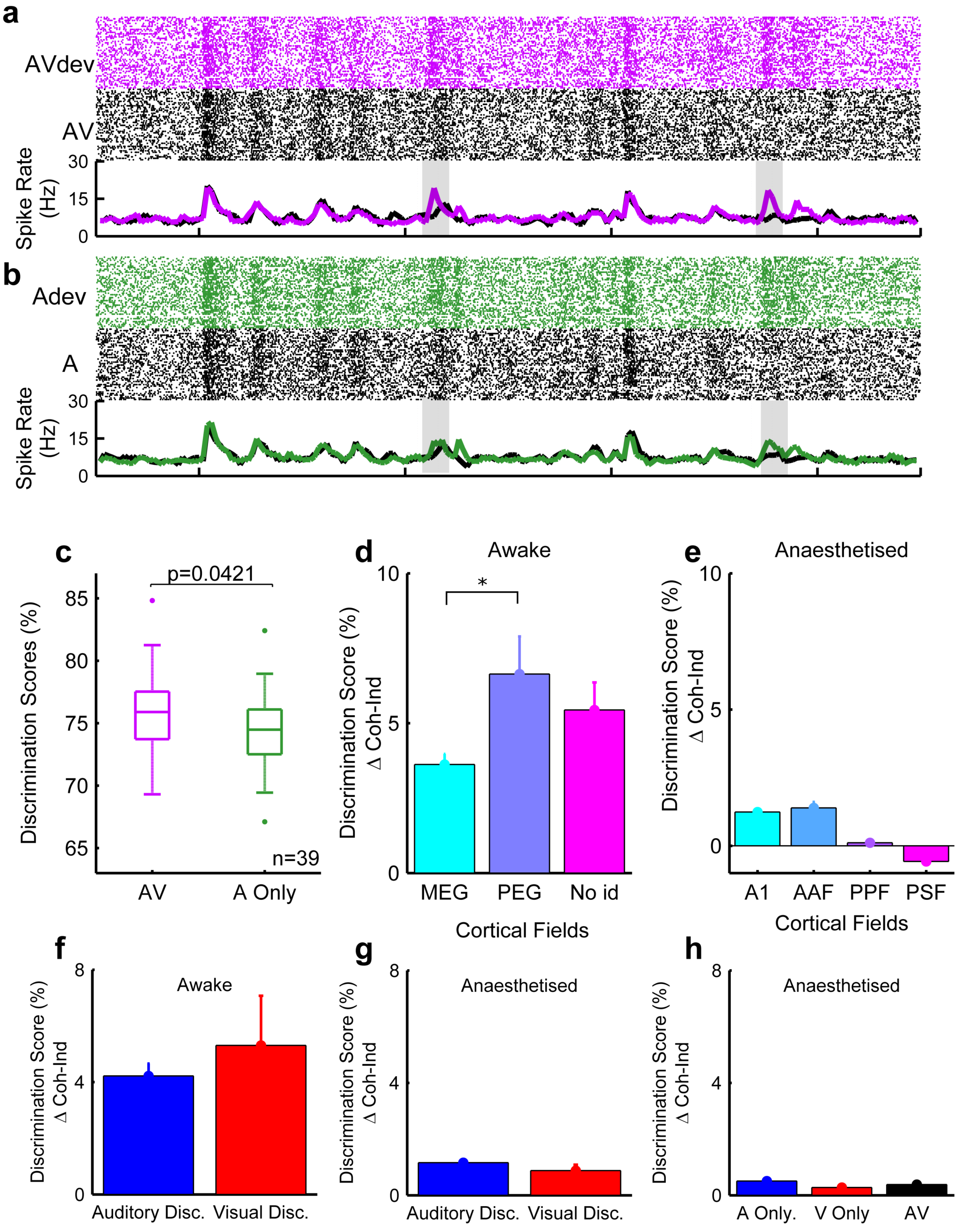

a,b, Rasters and PSTH for single stream stimuli containing deviants (top row, black) or without deviants (bottom row, purple/green). **a**, auditory stream presented with a temporally coherent visual stimulus, **b**, auditory stream presented in isolation. Grey panels indicate the timing of the timbre deviants. **c**, discrimination scores for detecting trials with deviants in them were significantly higher in AV trials than A only trials. Recordings were made in awake animals (# of animals =3, n=39 driven units,). Pairwise comparison t_{76} = 2.0676, p = 0.0421)

d,e, comparison of how visual temporal coherence influenced deviant encoding across cortical fields in awake data and anesthetised data. In awake data, a one-way ANOVA across cortical fields showed a significant effect of field ($F(2,165) = 2.6710$, $p= 0.0322$) with post-hoc comparisons indicating that the discrimination scores were greater in the PEG than MEG. However, there was no significant effect of field in anaesthetised data ($F(3,245) = 2.0627$, $p = 0.1057$)

f,g No differences were found in discrimination score between auditory discriminating and visual discriminating units in awake recordings ($F(1,100) = 0.2547$, $p = 0.6149$) or in anaesthetised recordings ($F(1,119) = 0.0689$, $p = 0.7933$).

h There were also no differences across different unit type in anaesthetised recording ($F(2,423) = 0.846$, $p = 0.9169$)

Supplemental Figure 7 (related to Figure 6):

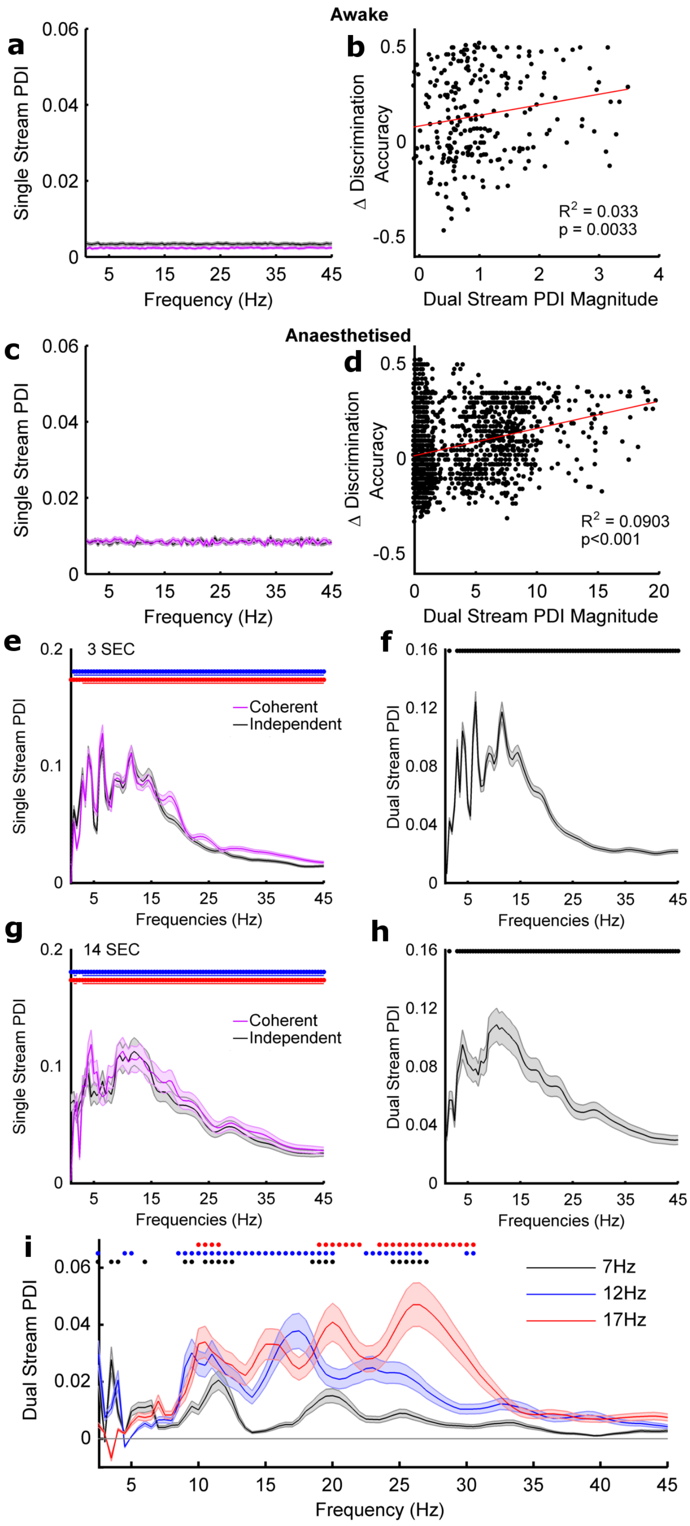

Power and phase dissimilarity analysis of the local field potential

a,c dual stream power discriminability index values for awake (a) and anesthetised datasets (c). In neither case was there any frequency whose power was significantly influenced by the visual stimulus identity. **b,d**, relationship between phase discriminability index (PDI) values measured in the dual stream condition and the VPI score. There is a weak correlation between the magnitude of the dual stream PDI values and VPI values.

During our initial analysis we observed that PDI values were higher in anesthetised animals than awake animals. In order to determine whether this was a difference due to behavioural state or simply an artefact of stimulus length for all of the analysis reported in this paper we restricted analysis of the anesthetised responses to the first three seconds of the stimulus. In **e-h** we explicitly compare the PDI values obtained for 3 second (**e,f**) and 14 second (**g,h**) single stream (**e,g**) and dual stream (**f,h**) stimuli. to match that recorded in the awake dataset. While phase coherence values were slightly higher for longer duration stimuli and hence at longer stimulus durations the ITPC profile and resulting PDI varied more smoothly with frequency. However at both durations phase values were significantly different from zero at all frequencies. The pattern of significant phase selectivity values was also preserved across stimulus durations. (**b, d**). Frequency points at which the single stream PDI value and dual stream PDI values

were similar in 3 second length (**a, b**) and 14 second length (**c, d**) Blue, red and black symbols indicate where the PDI was significant (pairwise t-test, a = 0.0012 with bonferoni correction).

i Dual stream stimuli were generated with three different amplitude modulation rates (<7Hz, as in the main experiment, <12Hz and <17Hz, values picked to avoid harmonics of 7 Hz) and responses to these were recorded in 92 units. Symbols indicate where the dual stream phase selectivity index was significant (pairwise t-test, $p<0.05$ with correction). In all three cases significant phase coherence is seen between 10Hz-11.5Hz, 19Hz-20Hz and 24-26 Hz.
